# ABO-incompatible living donor kidney transplantation failure due to acute blood group antibody-dependent rejection triggered by human parvovirus B19 infection: a case report and literature review

**DOI:** 10.3389/fmed.2023.1195419

**Published:** 2023-11-23

**Authors:** Lin-rui Dai, Xiao-hui Wang, Yi-bo Hou, Zhi-yu Zou, Song Chen, Wei-jie Zhang, Sheng Chang

**Affiliations:** ^1^Institute of Organ Transplantation, Tongji Hospital, Tongji Medical College, Huazhong University of Science and Technology, and Key Laboratory of Organ Transplantation, Ministry of Education, and NHC Key Laboratory of Organ Transplantation, and Key Laboratory of Organ Transplantation, Chinese Academy of Medical Sciences, Wuhan, China; ^2^Department of Urology, The First Affiliated Hospital of Henan University of Science and Technology, Luoyang, China

**Keywords:** ABO incompatibility, kidney transplantation, acute rejection, living donor, B19V infection, accommodation

## Abstract

**Background:**

With the improvement of immunosuppressive regimens, the success rate and availability of ABO-incompatible (ABO-i) kidney transplantation (KT) have gradually increased. However, the management of immunosuppression protocols and complications associated with ABO-i KT is complex. Here, we report a clinical case of ABO-i living donor KT with allograft dysfunction caused by acute blood group antibody-dependent rejection triggered by human parvovirus B19 (B19V).

**Case report:**

The ABO blood group of the recipient was O, and that of the donor was B. The recipient had high baseline anti-B antibody titers (IgM, 1:1024; IgG, 1:64). Before transplantation, he completed a desensitization protocol comprising plasma exchange, double-filtration plasmapheresis, and rituximab, which maintained a low blood group antibody level and resulted in successful transplantation. Two weeks after surgery, the recipient developed a B19V infection combined with acute T-cell-mediated rejection. After the anti-rejection regimen, acute rejection (AR) was successfully reversed, but B19V persisted. One week after AR stabilization, the patient experienced acute antibody-mediated rejection that was more severe and refractory, resulting in the loss of the transplanted kidney.

**Conclusion:**

Desensitization combined with immunosuppressants can lead to overimmunosuppression and cause various infections. Infections could break the accommodation state of the patient, thereby inducing AR and resulting in the loss of the transplanted kidney.

## 1 Introduction

Kidney transplantation (KT) is the best replacement therapy for patients with end-stage kidney disease. The shortage of deceased donor kidneys and long waiting times have led to a gradual increase in the proportion of living donor transplantations. Owing to the gradual maturation of appropriate immunological preparations to remove blood group antibodies and suppress their generation, ABO-incompatible (ABO-i) KT has been performed (1). ABO-i KT has been compared with ABO-compatible KT at several centers worldwide, and several studies have shown no significant differences in patient survival or graft survival of these two procedures (2). However, other studies have shown that ABO-i transplant recipients are at higher risk for serious infections and thrombotic microangiopathy (TMA) (3–5). Moreover, the incidence of antibody-mediated rejection (AMR) during the first 3 months is 58% for ABO-i transplant recipients (6). Among these patients, acute AMR (aAMR) is the primary cause of allograft failure with solid organ transplantation (7). Despite adequate desensitization therapy, anti-ABO antibodies may rebound after ABO-i KT.

If anti-ABO antibody titers are successfully neutralized during the first 3–4 weeks, then the transplanted kidney may establish a post-transplant state of accommodation, the graft will maintain its normal function in the presence of anti-ABO antibodies and complement, and anti-ABO antibody-mediated graft injury may not occur (8–10). However, excessive immunosuppression caused by high doses of immunosuppressants targeting T and B lymphocytes during desensitization and the early post-transplantation period increase the risk of infection of ABO-i KT recipients, which may break the accommodation state, thereby largely increasing the risk of AMR and eventually leading to serious graft impairment or even loss of function (4, 11). Although successful ABO-i KT has been performed, it is important to summarize the reasons for ABO-i living donor KT (LDKT) failure caused by complex factors.

## 2 Case description

**Figure 1:**
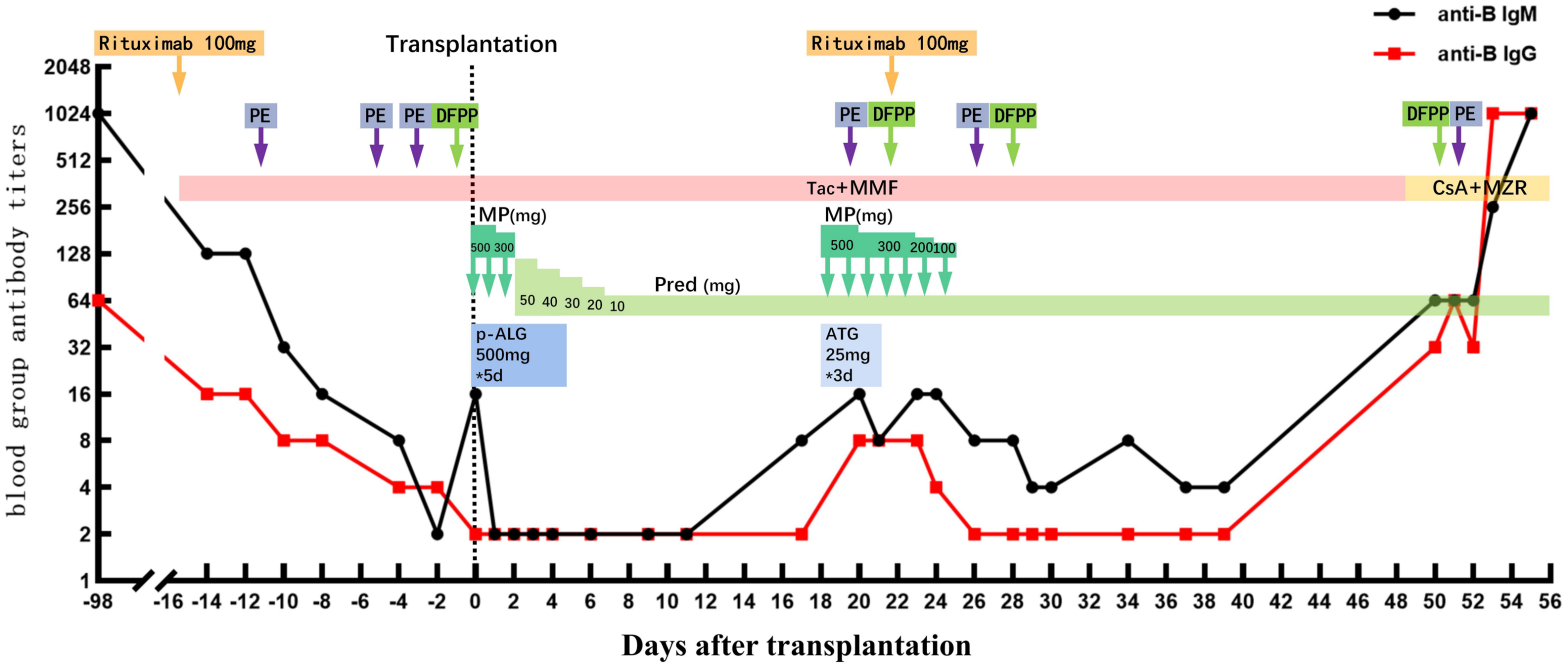
Blood group antibody titers and immunosuppressive regimen used before and after transplantation. The recipient’s ABO blood group was O and the donor’s blood group was B, and the recipient’s baseline anti-B antibody titers were 1:1,024 (anti-B IgM) and 1:64 (anti-B IgG), respectively. The recipient completed a pre-transplant desensitization protocol, whereafter anti-B antibody titers remained at low levels (equal or less than pre-transplant level 1:2) or declined to an undetectable level. Until POD 50, the recipient’s anti-B IgM and anti-B IgG had increased to 1:64 and 1:32, respectively, and then gradually increased to untreated pre-transplant levels. The pink rectangle indicates tacrolimus (Tac) and mycophenolate mofetil (MMF). The yellow rectangle indicates the change of cyclosporine (CsA) and mizoribine (MZR) on POD49. The orange arrows indicate days of using rituximab. The purple arrows indicate days of using plasma exchange (PE). The grass green arrows indicate days of using double filtration plasmapheresis (DFPP). The dark green arrows indicate days and dose of using methylprednisolone (MP). The green rectangle indicates days and dose of using prednisone (Pred), staring at 50 mg/d and then prednisone tapered by 10 mg every other day to 10 mg/d for maintenance. The dark blue and light blue boxes indicate the dose and days of using anti-human T lymphocyte porcine immunoglobulin (p-ALG) and rabbit anti-human thymocyte immunoglobulin (ATG). The black dashed line indicates the day of transplantation.

The LDKT recipient was a 34-year-old man (height, 174 cm; weight, 68 kg; blood group, O). The patient’s primary nephropathy was chronic glomerulonephritis, and he had been on regular dialysis for >5 years. Before transplantation, donor-specific anti-human leukocyte antigen (HLA) antibodies and panel-reactive antibodies (PRA) were negative. The recipient’s mother was the donor (age, 64 years; height, 157 cm; weight, 60 kg; blood group, B). Complement-dependent cytotoxicity cross-matches and flow cytometry cross-matches were negative. The recipient underwent ABO-i and HLA-A, HLA-B, HLA-C, HLA-DR, HLA-DP, and HLA-DQ 4/12 mismatched KT. The baseline anti-B antibody titers of the recipient before transplantation were 1:1,024 (IgM) and 1:64 (IgG) (Figure 1). The donor volunteered to donate a kidney to her son and provided written informed consent. This study was approved by the ethics committees of Tongji Hospital, Tongji Medical College, Huazhong University of Science and Technology, and the Health Commission of Hubei Province.

The recipient was admitted to the hospital 3 weeks before surgery. We formulated and started a desensitization protocol before transplantation. First, the recipient was intravenously administered 100 mg of rituximab on day 15 before surgery. Immediately thereafter, tacrolimus (TAC) 0.1 mg/kg/day and mycophenolate mofetil (MMF) 1,500 mg/day were administered orally in two doses. Subsequently, the immunosuppressive doses were adjusted according to the TAC trough level and the area under the curve at the time of MMF. The TAC trough level before surgery fluctuated around 8 ng/mL, and the area under the curve at the time of MMF was controlled at 60 μg/h/mL, which inhibited the activation and proliferation of T and B lymphocytes and the levels of blood group antibodies. Simultaneously, three courses of plasma exchange (PE) and one course of double-filtration plasmapheresis (DFPP) were administered to remove blood group antibodies. According to the protocol, transplantation was considered when anti-B titers were less than 1:16 for 2 consecutive days. On the day of transplantation, the anti-B titers were reduced to 1:16 (IgM) and 1:2 (IgG) (Figure 1).

The donor’s left kidney was subjected to warm ischemia for 1 min and promptly transplanted to the recipient. The allograft was functionally perfect after reperfusion. Perioperative immunosuppression included induction therapy with anti-human T-lymphocyte porcine immunoglobulin and methylprednisolone and triple maintenance therapy with TAC, MMF, and prednisone (intravenous porcine immunoglobulin, 500 mg, days 0–4; intravenous methylprednisolone, 500 mg, days 0–2; and oral prednisone, 50 mg, day 3). Oral prednisone was subsequently tapered by 10 mg every day to 10 mg/day for maintenance. The preoperative dosing regimens of TAC and MMF were continued. The postoperative TAC trough level was maintained at approximately 8 ng/mL. The renal function of the recipient improved postoperatively. Serum creatinine decreased from 1,125 μmol/L before surgery to 159 μmol/L on postoperative day (POD) 11. The IgG and IgM anti-B titers remained low throughout the postoperative course (1, 2). The patient was discharged after an uneventful course on POD 12.

**Figure 2:**
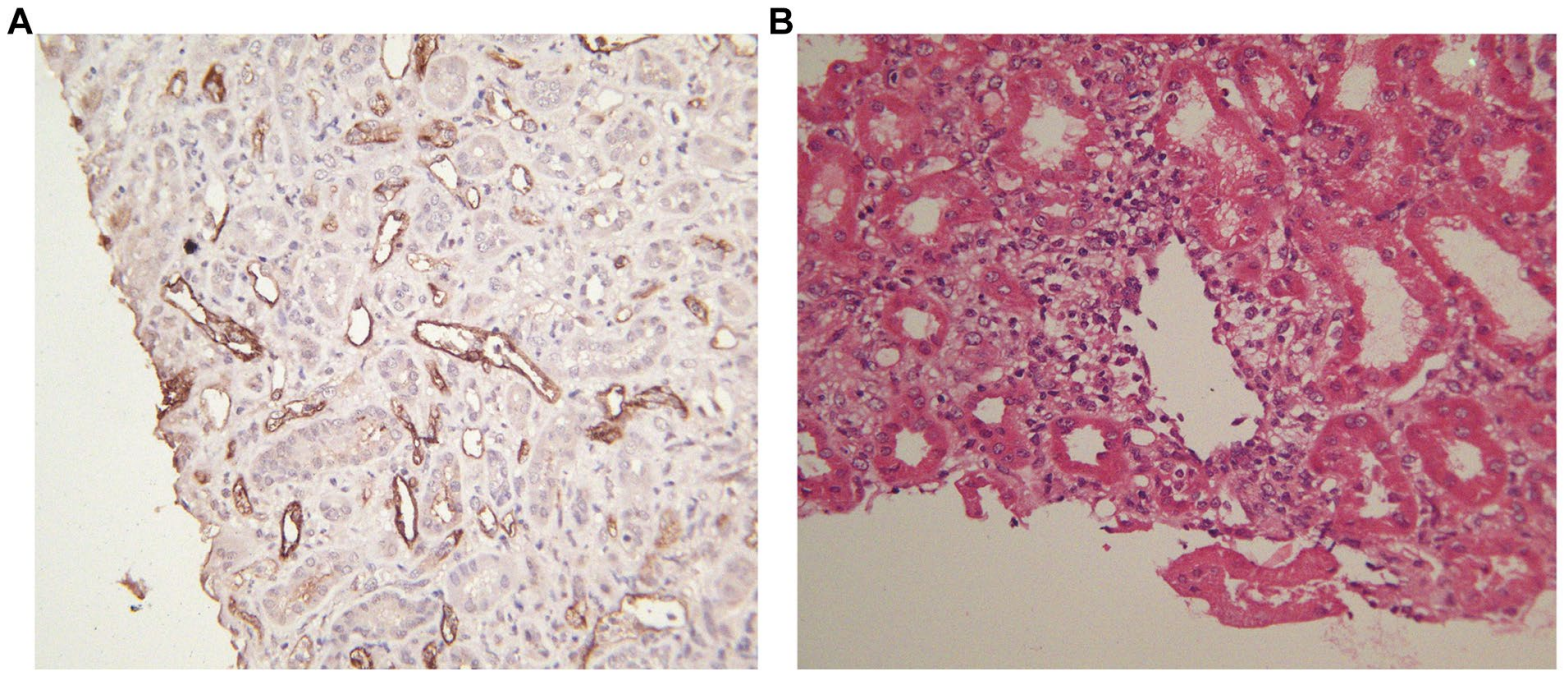
Allograft biopsy specimen obtained on POD 18. **(A)** Immunohistochemical staining: C4d was diffusely positive (×200). **(B)** One micro-vein presented with venous endotheliitis, mild renal interstitial edema, patchy and diffuse infiltration of lymphocytes in the interstitium, patchy and mild renal tubulitis, and a few peritubular capillaritis; the interstitial matrix of renal tissue did not show hyperplasia and tubular atrophy; mild water degeneration of renal tubular epithelial cells and no necrosis of renal tubular epithelial cells (H.E, ×400).

**Figure 3:**
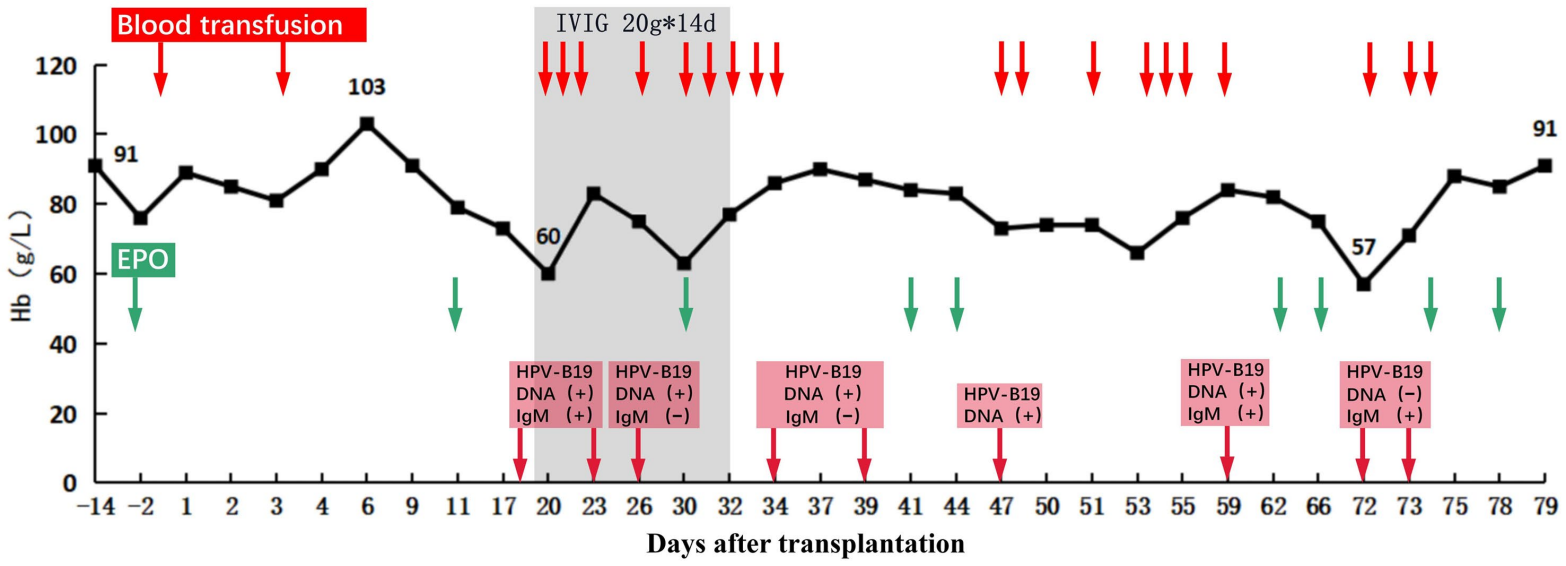
Hemoglobin levels before and after transplantation. After transplantation, the recipient’s hemoglobin increased to 103 g/L at the highest level and then showed a progressive decrease in hemoglobin without obvious cause, which reached as low as 60 g/L on POD 19. After treatment, the recipient’s hemoglobin (Hb) recovered and remained stable. The red arrows indicate days of using transfusion. The Green arrows indicate days of using recombinant human erythropoietin (EPO). The red squares indicate the results of HPV-B19 DNA and IgM testing, and the arrow points to the time of testing.

On POD 17, the patient presented with fever and a progressive decrease in hemoglobin level to 73 g/L. Excluding other causes, the patient was readmitted to the hospital because of high suspicion of human parvovirus B19 (B19V) causing pure red cell aplasia. His serum creatinine level had increased to 514 μmol/L and was accompanied by positive B19V IgM and DNA and a sharp decrease in urine volume to 950 mL/day. The TAC trough concentration was 2.9 ng/mL; however, there was no increase in antibody titers (anti-B IgM and IgG titers of 1:8 and 1:2, respectively). Furthermore, graft perfusion had reduced, and the arterial resistance index had increased on Doppler ultrasonography. Therefore, the patient was preliminarily considered to have B19V and acute rejection (AR). To further assess the condition of the allograft, we immediately performed a graft biopsy. Pathological findings confirmed grade IA mild acute T-cell-mediated rejection (aTCMR) (Banff 2019 grade IA, i1, t1, g0, v0, ci0, ct0, cg0, cv0, ptc1, g + ptc = 1, ah0, mm0, i-IFTA0, t-IFTA0, and diffusely positive C4d3) (Figure 2A), with only a few minor peritubular capillaritis (ptc1) in the allograft biopsy sample and no manifestation of glomerulonephritis (Figure 2B). The B19 DNA in paraffin-embedded kidney tissue was amplified by nested PCR, and the results were negative. Methylprednisolone (500 mg for 2 days, 300 mg for 3 days, 200 mg for 1 day, and 200 mg for 1 day) and rabbit anti-human thymocyte immunoglobulin pulse therapy (25 mg for 3 days) were immediately administered. The patient promptly received intravenous immunoglobulin (IVIG) at 20 g/day for 14 days. Red blood cell transfusion was simultaneously performed for pure red cell aplasia. Subsequently, hemoglobin levels gradually increased (Figure 3).

**Figure 4:**
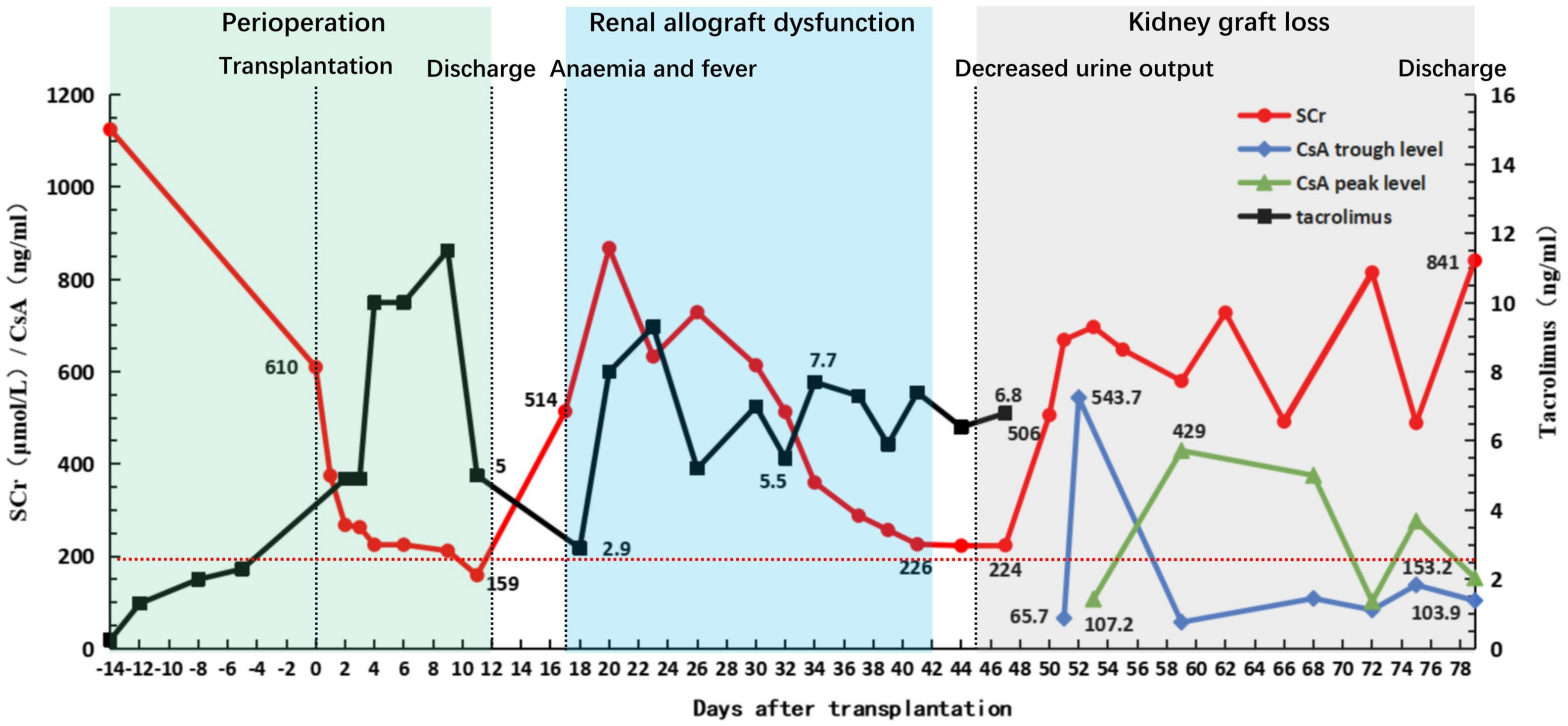
Clinical course of the patient. Graft function and fluctuation of immunosuppressant concentration before and after transplantation. The green square indicates the patient’s first hospitalization and discharge with good recovery of graft function (with serum creatinine (SCr) rapidly declining to 200 μmol/L within 10 days post-transplantation). The blue square indicates that the patient was readmitted for treatment due to HPV-B19 infection and aTCMR, after which the patient improved following a series of treatments. The gray square indicates the patient’s injury of graft function following a fierce anti-blood group antibody-mediated acute humoral rejection until the graft was lost and the patient was discharged again after resuming regular hemodialysis therapy.

On POD 19, the anti-B IgM and IgG titers were 1:16 and 1:8, respectively, showing a rebound trend. To avoid injury caused by anti-B antibodies that trigger aAMR, the patient received 100 mg rituximab intravenously and four courses of PE or DFPP (Figure 1). The antibody titer did not increase continuously. The urine volume gradually returned to more than 2,000 mL/day, and the serum creatinine level decreased to 226 μmol/L. The TAC trough concentration was 7.4 ng/mL (Figure 4), and the anti-B titers decreased to 1:4 (IgM) and 1:2 (IgG). Allograft perfusion was adequate, and the arterial resistance index was within the normal range, as evaluated by Doppler ultrasonography. Therefore, we believe that aTCMR was reversed after treatment.

As the patient’s condition improved, we assumed that the graft function improved as well. He recovered well; however, the urine volume gradually decreased on POD 45 and the serum lactate dehydrogenase (LDH) level significantly increased to 269 U/L and the platelet level was 40 × 109/L (the preoperative serum LDH level was 126 U/L). Platelet-boosting therapy was given at this time. Repeated qualitative tests to determine B19V DNA yielded positive results. TAC was replaced with cyclosporine A as antiviral therapy on POD 48. On POD 50, the serum creatinine and serum LDH levels increased sharply to 506 mol/L and 356 U/L, the platelet level was 83 × 10^9^/L, and anti-B titers increased to 1:64 (IgM) and 1:32 (IgG). Doppler ultrasonography revealed swelling, enhanced parenchymal echo, and sparse blood flow in the transplanted kidney; therefore, renal artery and vein embolisms were ruled out. Donor-specific anti-HLA antibodies and PRA tests yielded negative results. This indicated that the subsequent AR was more aggressive. The patient immediately underwent two courses of PE or DFPP to remove the antibodies. We recommended another renal allograft biopsy, but the patient refused. On POD 52, Doppler ultrasonography revealed no blood supply to the renal allograft. Subsequently, the serum creatinine level increased to 697 μmol/L, and the anti-B antibody titers promptly increased (IgM, 1:1024; IgG, 1:256) (Figure 1). As the calcineurin inhibitors and lymphocyte function indicators were within reasonable ranges, we concluded that the patient had experienced an acute blood group antibody-mediated humoral rejection of the allograft and that kidney function had deteriorated drastically to an irreversible state. The patient had received salvage therapy for the transplanted kidney and returned to conventional hemodialysis.

## 3 Discussion

Here, we report a clinical case of renal allograft loss due to severe acute humoral rejection mediated by anti-blood group antibody after ABO-i living-related renal transplantation, probably triggered by B19V infection. Based on our current knowledge, no similar cases have been reported to date. Both inadequate and excessive immunosuppression can result in cascade reactions such as rejection and infection. The patient featured higher baseline anti-B titers (IgM 1:1024; IgG 1:64), which undoubtedly increased the frequency of preoperative PE or DFPP and the intensity of immunosuppression, and therefore, the patient lost more immunoglobulins (12). Meanwhile, high-intensity immunosuppression during desensitization therapy and in the early post-transplant period increased the risk of infection in ABO-i KT recipients. Furthermore, there has been controversy regarding the high AMR rate and early graft loss in recipients with high baseline blood group antibody titers (13). However, in any case, immunosuppressive regimens used to remove high baseline blood group antibody titers could result in a higher risk of infection (14, 15). High baseline anti-B titers in patients may contribute to the loss of the transplanted kidney. One week after transplantation, the recipient experienced decreased hemoglobin levels with no apparent trigger. Thereafter, B19V was diagnosed, implying excessive immunosuppression. Because B19V mainly manifests as pure red cell aplasia (16), the patient experienced markedly decreased hemoglobin and a significantly decreased TAC trough concentration. AR may be related to insufficient immune strength caused by the reduction and replacement of immunosuppression after B19V infection (17, 18).

Postoperative week 2 involves a high incidence of AR and is a high-risk period for ABO-i KT recipients (19). Results of the pathological biopsy performed 2 weeks after the operation in this recipient indicated mild aTCMR, with peritubular capillaritis with diffuse C4d deposits (C4d3) in the kidney allograft, which usually predict aAMR. Despite diffuse C4d peritubular capillary deposition, the transplanted kidney can establish a state of post-transplant accommodation in which the allograft maintains the normal long-term function of the host without AR injury (11, 20). Therefore, C4d staining cannot be used to diagnose AMR in ABO-i KT recipients (21, 22). Ultimately, we adopted efficacious anti-rejection therapies that successfully reversed aTCMR, and the recipient recovered from the initial renal graft function impairment.

aAMR is a major cause of graft dysfunction. It contributes to solid organ transplantation failure (7) and is mediated by preexisting and *de novo* antibodies, including major HLA, minor non-HLA, and A/B blood group antibodies. Two types of aAMR with ABO-i KT have been suggested (13). The first type of aAMR is caused by repeat sensitization to ABO antigens. If antibody production, including that of memory lymphocytes, is insufficiently inhibited, then ABO antigens induce a secondary immune response. This leads to an explosive production of antibodies, culminating in violent aAMR, which usually manifests as an increase in IgG titers accompanied by a parallel increase in IgM titers (23). When this occurs, there is no response to currently available therapies, ultimately resulting in graft loss. The other type of aAMR is attributed to primary sensitization caused by ABO antigens. This type of aAMR is associated with increased serum IgM titers. This type of aAMR progresses more slowly and is less severe. Patients with this type of aAMR respond well to treatment and have good graft survival and renal function (24). However, it is unclear which type of ABO antibody (IgM or IgG) is more clinically important in ABO-i KT. The antibody response to non-protein antigens primarily depends on IgM production. IgM is more capable of complement fixation and activation than IgG. Studies that relied on the molecular characteristics of ABO antigens and evaluated the clinical significance of anti-A/B IgM and IgG in ABOi KT suggested that anti-A/B IgM might play a critical role in AMR (25). This case appears to be consistent with a type of aAMR caused by repeat sensitization to ABO antigens, which may have been the ultimate cause of graft loss.

However, a condition known as accommodation during the post-transplantation period, which can be broadly defined as the absence of allograft injury despite the presence of anti-donor antibodies in the recipient, has been described. It is generally believed that the ABOi KT enters this stabilization period after 2 weeks. There is no blood group antibody rebound or AMR, and kidney function remains stable for a long time (11). In ABOi transplants, the rebound of A/B blood group antibodies after transplantation without any pathological manifestation of rejection is indicative of the state of accommodation, and similar phenomena with xenograft transplants have been observed (26, 27). The second occurrence of rejection in the recipient was characterized by rapid and severe acute blood group antibody-dependent rejection. We considered this to be related to the collapse of the postoperative accommodation state (10). Accommodation breakdown may be associated with high preoperative baseline antibody titers and postoperative B19V onset (28).

B19V has a tropism for renal endothelium (16) and may have direct cytopathic effects on glomerular epithelial cells or endothelial cells and glomerular deposition of immune complexes (29, 30). Kidney injury caused by B19V often results in pathological manifestations, such as focal segmental glomerulosclerosis, collapsing glomerulopathy, endocapillary proliferative glomerulonephritis, and thrombotic microangiopathy (31–35). There are no specific antiviral therapies for B19V. Reductions in the intensity of immunosuppression and IVIG constitute the cornerstone of therapeutic management of B19V after renal transplantation (36). However, both treatments may cause a rebound of blood antibody titers (37, 38). Inadequate immunosuppression may induce reactivation of T and B lymphocytes, thus inducing *de novo* blood group antibodies and resulting in titer rebound. However, there is evidence of a certain level of blood group antibodies remaining in IVIG because of manufacturing processes, which may lead to increased blood group antibody levels in the ABO-i KT recipients after infusion (38). This recipient was treated with IVIG products from two manufacturers; therefore, we retrospectively measured the anti-B titers of the same batches of IVIG from these manufacturers. However, both had low antibody titers (Shandong Taibang Biological Products Co., Ltd.: IgM <1:2 and IgG = 1:4; Harbin Baishi Huike Biopharmaceutical Co., Ltd.: IgM < 1:2 and IgG = 1:2). Additionally, because there was no significant increase in the recipient’s blood group antibody titers during IVIG infusion, the sharp rebound in anti-B titers is unlikely to have originated from IVIG infusion.

After our first successful reversal of aTCMR, we reduced the frequency of blood group antibody monitoring. The serum LDH level was more than twice that of the pre-transplantation level. The recipient also experienced a decreased platelet count. However, this was not considered during this case management. We overlooked the fact that ABO-i KT is more likely to cause TMA, which is a rare but severe complication after transplantation (37). TMA is characterized by rapidly progressive renal graft dysfunction and often has a poor prognosis. The diagnostic criteria for TMA include a postoperative lactate dehydrogenase level more than 2-fold higher than the baseline level, anemia or the need for transfusion therapy, and decreased platelet count (<50 × 10^9^/L or < 50% reduction). Additionally, viral infections and AMR are risk factors for TMA (29, 39–41). However, we did not consider the possibility of this adverse event. aAMR is a common and important cause of *de novo* TMA after transplantation, and sometimes both co-exist (42). As TMA was included in the diagnosis of aAMR and according to the Banff criteria, microvascular inflammation is also a criterion of aAMR (43). A study by Tasaki et al. revealed that all TMA cases were biopsy-proven aAMR and that TMA occurred with increasing antibody titers in these recipients whose grafts showed aAMR (3). However, we speculated that allograft loss in this recipient may have been linked to TMA because of the lack of novel transplant biopsy evidence to support this inference.

It has been hypothesized that B19V infection can lead to allograft dysfunction and acute or chronic allograft rejection through direct cytopathic effects and immune responses (32, 44–46). It is difficult to determine a causal relationship between B19V and allograft rejection or dysfunction. Nevertheless, it has been suggested that B19V targets the endothelium, which acts as an antigen-presenting cell during infection, with overexposure to major histocompatibility complex class II and the activation of acquired immunity, ultimately leading to humoral reactions, which lead to AMR and the production of donor-specific anti-HLA antibodies (29, 47, 48). Some researchers believe that endothelial cell injury caused by B19V and the subsequent sensitization of the glomerular endothelium may increase the incidence of AMR (41). Reducing the impact of the direct and indirect effects of B19V after renal transplantation is important to improve graft survival and function.

Cases of B19V after KT with allograft loss or dysfunction and rejection have been reported (30, 46, 49–51); however, literature on this aspect is limited. The first report of B19V after renal transplantation was published in 1986 (52). Zolnourian et al. (31) implicated B19V as a cause of AR and graft loss. To the best of our knowledge, there have been two cases similar to our case in China (unpublished). AMR induced renal function loss caused by persistent B19V after renal transplantation that pathologically manifested as TMA. Persistent B19V may increase the AMR incidence and is associated with a higher risk of chronic graft dysfunction (30, 53). The characteristics of the cases of B19V-triggered rejection episodes are presented in Table 1. Our case of ABOi KT involved allograft dysfunction secondary to aAMR triggered by B19V and highlights the importance of including B1V9 in the diagnostic evaluation of graft failure after KT. This recipient was infected with B19V in the early postoperative period and aTCMR occurred at the same time. The treatments for AR and B19V infection are diametrically opposed, with the former requiring stronger immunosuppressive treatment and the latter requiring less immunosuppressive treatment. Although aTCMR was successfully reversed, the B19V infection continued, which led to the state of accommodation to break down and trigger aAMR, which resulted in the rapid deterioration of the transplanted renal function of the recipient and graft loss.

**Table 1:** Characteristics of the cases of B19V-triggered rejection episodes.

Study	Year	Country	Patients (n)	B19V detection	Clinical manifestation	Renal allograft pathology	Patient outcome
Barzon et al. (30)	2009	Italy	Kidney transplant patients (7)	PCR	AR (7) fever, rash, and hyporegenerative anemia (1)	AR (7) High copy numbers of B19V DNA (7) C4d-positive aAMR (1) TMA (1)	Graft survival (6) Acute graft dysfunction (1)
Zolnourian et al. (31)	2009	Northern Ireland.	Kidney transplant patients (1)	lgM, lgG, and PCR	AR (1)	Acute vascular rejection (1)	Graft failure (1)
Murer et al. (35)	2000	Italy	Kidney transplant patients (1)	lgM, lgG, and PCR	AR, fever, fatigue and arthralgia, aplastic anemia, and thrombocytopenia (1)	TMA (1) Histologic rejection (1)	Graft survival (1)
Eid et al. (49)	2006	US	Simultaneous kidney and pancreas transplant patients (1)	lgM and lgG	Chronic rejection, PRCA, and leukopenia (1)	Chronic rejection (1)	Graft failure (1)
Knysak et al. (50)	2020	Poland	Second kidney transplant patients (1)	lgM, lgG, and PCR	AR and PRCA (1)	aAMR (1) acute tubular necrosis (1) BKV infection (1)	Graft survival (1)
Ki et al. (51)	2005	Korea	Kidney transplant patients (7)	PCR	AR (7) PRCA (2)	AR (7)	Graft survival (6) Graft failure (1)
Bertazza et al. (53)	2023	Italy	Kidney transplant patients (15)	PCR	AR (15)	aAMR (8) aTCMR (7)	Graft survival (15)

AR, acute rejection; PRCA, pure red cell aplasia; TMA, thrombotic microangiopathy; aAMR, acute antibody-mediated rejection; aTCMR, acute T-cell-mediated rejection.

In conclusion, although ABOi KT has been widely developed, managing immunosuppressive regimens and postoperative complications is more complex than managing ABO-compatible KT. Both inadequate and excessive immunosuppression could induce rejection and infection, respectively. The treatment of infections combined with rejection and immunosuppressive therapy is challenging. The risk of graft loss increases when the accommodation status after transplantation is broken or aAMR is triggered by repeat sensitization to ABO antigens. These results should be comprehensively analyzed, and viral infections caused by excessive immunosuppression should be avoided. When blood group antibody titers rebound, it should be determined whether the recipient was in the accommodation state or whether aAMR was triggered by ABO antigens. The latter may lead to the irreversible loss of the kidney allograft. Based on this case, we believe that the postoperative hospitalization period should be appropriately extended for ABOi KT recipients to allow them to safely overcome the postoperative high-risk period. The blood group antibody titers and immune function status should be monitored for 3 months after surgery. Patients with high baseline blood group antibody titers, anti-A/B titers, and various virological parameters, especially B19V, should be closely monitored after transplantation.

## Data availability statement

The original contributions presented in the study are included in the article/Supplementary material, further inquiries can be directed to the corresponding author.

## Ethics statement

The donor-recipient relationship is mother-to-son. The donor volunteered to donate a kidney for her son, and all of the donor’s immediate family members signed a written informed consent form before the operation. The living-related kidney transplantation was approved by the Ethics Committee of Tongji Hospital, Tongji Medical College, Huazhong University of Science and Technology, and the Ethics Committee of Health Commission of Hubei Province, China. Written informed consent to participate in this study was provided by the participants. Written informed consent was obtained from the individual(s) for the publication of any potentially identifiable images or data included in this article.

## Author contributions

SoC, W-jZ, ShC, and Z-yZ performed the surgery. L-rD wrote the manuscript. SC and X-hW revised and edited the manuscript. Y-bH performed the flow. All authors participated in patient management and data collection and contributed to the article and approved the submitted version.

## References

[1] de Weerd AE Betjes M. ABO-incompatible kidney transplant outcomes: a meta-analysis. Clin J Am Soc Nephrol. (2018) 13:1234–43. doi: 10.2215/CJN.00540118, PMID: 30012630 PMC6086717

[2] Kosoku A Uchida J Nishide S Kabei K Shimada H Iwai T. ABO-incompatible kidney transplantation as a renal replacement therapy—a single low-volume center experience in Japan. PLoS One. (2018) 13:e208638. doi: 10.1371/journal.pone.0208638, PMID: 30596663 PMC6312268

[3] Tasaki M Saito K Nakagawa Y Imai N Ito Y Yoshida Y. Analysis of the prevalence of systemic *de novo* thrombotic microangiopathy after ABO-incompatible kidney transplantation and the associated risk factors. Int J Urol. (2019) 26:1128–37. doi: 10.1111/iju.14118, PMID: 31587389

[4] Ko Y Kim JY Kim S Kim DH Lim SJ Shin S. Acute rejection and infectious complications in ABO- and HLA-incompatible kidney transplantations. Ann Transpl. (2020) 25:e927420. doi: 10.12659/AOT.927420, PMID: 33020465 PMC7547531

[5] Hirzel C Projer L Atkinson A Surial B Mueller NJ Manuel O. Infection risk in the first year after ABO-incompatible kidney transplantation: a nationwide prospective cohort study. Transplantation. (2022) 106:1875–83. doi: 10.1097/TP.0000000000004109, PMID: 35389968

[6] Ali T Broering D Aleid H Brockmann J Alhumaidan H Hammad E. ABO incompatible kidney transplantation: the Saudi experience. Saudi J Kidney Dis Transpl. (2019) 30:655–62. doi: 10.4103/1319-2442.261340, PMID: 31249230

[7] Kenta I Takaaki K. Molecular mechanisms of antibody-mediated rejection and accommodation in organ transplantation. Nephron. (2020) 144:2–6. doi: 10.1159/000510747, PMID: 33238285

[8] Maritati F Bini C Cuna V Tondolo F Lerario S Grandinetti V. Current perspectives in ABO-incompatible kidney transplant. J Inflamm Res. (2022) 15:3095–103. doi: 10.2147/JIR.S360460, PMID: 35642217 PMC9148605

[9] Park S Lee J Jang JY Ryu J Kim DJ Chang SJ. Induction of accommodation by anti–complement component 5 antibody-based immunosuppression in ABO-incompatible heart transplantation. Transplantation. (2019) 103:e248–55. doi: 10.1097/TP.0000000000002808, PMID: 31461745

[10] Jeon HJ Lee JG Kim K Jang JY Han SW Choi J. Peripheral blood transcriptome analysis and development of classification model for diagnosing antibody-mediated rejection vs accommodation in ABO-incompatible kidney transplant. Am J Transplant. (2019) 20:112–24. doi: 10.1111/ajt.15553, PMID: 31373158

[11] De Mattos G Barbosa M Cascalho M Platt JL. Accommodation in ABO-incompatible organ transplants. Xenotransplantation. (2018) 25:e12418. doi: 10.1111/xen.12418, PMID: 29913044 PMC6047762

[12] Hanaoka A Naganuma T Kabata D Takemoto Y Uchida J Nakatani T. Selective plasma exchange in ABO-incompatible kidney transplantation: comparison of substitution with albumin and partial substitution with fresh frozen plasma. Sci Rep. (2020) 10:10. doi: 10.1038/s41598-020-58436-2, PMID: 31996738 PMC6989510

[13] Takahashi K. Recent findings in ABO-incompatible kidney transplantation: classification and therapeutic strategy for acute antibody-mediated rejection due to ABO-blood-group-related antigens during the critical period preceding the establishment of accommodation. Clin Exp Nephrol. (2007) 11:128–41. doi: 10.1007/s10157-007-0461-z, PMID: 17593512

[14] Ray DS Thukral S. ABO-incompatible renal transplantation with high antibody titer: a case report. Am J Case Rep. (2017) 18:1073–6. doi: 10.12659/AJCR.905633, PMID: 28983073 PMC5642648

[15] Won D Choe W Kim HJ Kwon SW Han DJ Park SK. Significance of isoagglutinin titer in ABO-incompatible kidney transplantation. J Clin Apher. (2014) 29:243–50. doi: 10.1002/jca.21312, PMID: 24375675

[16] Eid AJ Ardura MI. Human parvovirus b19 in solid organ transplantation: guidelines from the American society of transplantation infectious diseases community of practice. Clin Transpl. (2019) 33:e13535. doi: 10.1111/ctr.13535, PMID: 30973192

[17] Rosado-Canto R Carrillo-Pérez DL Jiménez JV Cuellar-Rodríguez JM Parra-Avila I Alberú J. Treatment strategies and outcome of parvovirus b19 infection in kidney transplant recipients: a case series and literature review of 128 patients. Rev Invest Clin. (2019) 71:71. doi: 10.24875/RIC.19002921 31448778

[18] Krishnan P Ramadas P Rajendran P Madhavan P Alex A Jayaschandran V. Effects of parvovirus b19 infection in renal transplant recipients: a retrospective review of three cases. Int J Angiol. (2015) 24:87–92. doi: 10.1055/s-0034-1371759, PMID: 26060378 PMC4452608

[19] Becker LE Süsal C Morath C. Kidney transplantation across HLA and ABO antibody barriers. Curr Opin Organ Tran. (2013) 18:445–54. doi: 10.1097/MOT.0b013e3283636c20, PMID: 23838650

[20] Cohen D Colvin RB Daha MR Drachenberg CB Haas M Nickeleit V. Pros and cons for C4d as a biomarker. Kidney Int. (2012) 81:628–39. doi: 10.1038/ki.2011.497, PMID: 22297669 PMC3771104

[21] Hruba P Krejcik Z Stranecky V Maluskova J Slatinska J Gueler F. Molecular patterns discriminate accommodation and subclinical antibody-mediated rejection in kidney transplantation. Transplantation. (2019) 103:909–17. doi: 10.1097/TP.0000000000002604, PMID: 30801516

[22] van Sandwijk MS Klooster A Ten BI Diepstra A Florquin S Hoelbeek JJ. Complement activation and long-term graft function in ABO-incompatible kidney transplantation. World J Nephrol. (2019) 8:95–108. doi: 10.5527/wjn.v8.i6.95 31662955 PMC6817790

[23] Morath C Zeier M Dohler B Opelz G Susal C. ABO-incompatible kidney transplantation. Front Immunol. (2017) 8:234. doi: 10.3389/fimmu.2017.00234 28321223 PMC5338156

[24] Takahashi K Saito K. ABO-incompatible kidney transplantation. Transplant Rev. (2013) 27:1–8. doi: 10.1016/j.trre.2012.07.003 22902167

[25] Kim H Choe W Shin S Kim YH Han DJ Park SK. ABO-incompatible kidney transplantation can be successfully conducted by monitoring IgM isoagglutinin titers during desensitization. Transfusion. (2020) 60:598–606. doi: 10.1111/trf.15672 31957888

[26] Bentall A Jeyakanthan M Braitch M Cairo CW Lowary TL Maier S. Characterization of ABH-subtype donor-specific antibodies in ABO-a-incompatible kidney transplantation. Am J Transplant. (2021) 21:3649–62. doi: 10.1111/ajt.16712, PMID: 34101982 PMC8597088

[27] Wang J Feng H Zhang C Zhong S Wang L Zhu L. Establishment of a hyperacute rejection model of ABO-incompatible renal transplantation in nonhuman primates. Front Immunol. (2021) 12:807604. doi: 10.3389/fimmu.2021.807604, PMID: 34970278 PMC8712559

[28] Tobian AA Shirey RS Montgomery RA Cai W Haas M Ness PM. ABO antibody titer and risk of antibody-mediated rejection in ABO-incompatible renal transplantation. Am J Transplant. (2010) 10:1247–53. doi: 10.1111/j.1600-6143.2010.03103.x 20420632

[29] Waldman M Kopp JB. Parvovirus B19 and the kidney. Clin J Am Soc Nephrol. (2007) 2:S47–56. doi: 10.2215/CJN.01060307, PMID: 17699510

[30] Barzon L Murer L Pacenti M Biasolo MA Della VM Benetti E. Investigation of intrarenal viral infections in kidney transplant recipients unveils an association between parvovirus B19 and chronic allograft injury. J Infect Dis. (2009) 199:372–80. doi: 10.1086/596053 19099488

[31] Zolnourian ZR Curran MD Rima BK Coyle PV O’Neill HJ Middleton D. Parvovirus B19 in kidney transplant patients. Transplantation. (2000) 69:2198–202. doi: 10.1097/00007890-200005270-00043 10852625

[32] Dupont PJ Manuel O Pascual M. Infection and chronic allograft dysfunction. Kidney Int. (2010) 78:S47–53. doi: 10.1038/ki.2010.423 21116318

[33] Ardalan MR Shoja MM Tubbs RS Esmaili H Keyvani H. Postrenal transplant hemophagocytic lymphohistiocytosis and thrombotic microangiopathy associated with parvovirus B19 infection. Am J Transplant. (2008) 8:1340–4. doi: 10.1111/j.1600-6143.2008.02244.x 18522549

[34] Moudgil A Nast CC Bagga A Wei L Nurmamet A Cohen AH. Association of parvovirus B19 infection with idiopathic collapsing glomerulopathy. Kidney Int. (2001) 59:2126–33. doi: 10.1046/j.1523-1755.2001.00727.x, PMID: 11380814

[35] Murer L Zacchello G Bianchi D Dall’amico R Montini G Andreetta B. Thrombotic microangiopathy associated with parvovirus B19 infection after renal transplantation. J Am Soc Nephrol. (2000) 11:1132–7. doi: 10.1681/ASN.V1161132 10820178

[36] Qiu J Soderlund-Venermo M Young NS. Human parvoviruses. Clin Microbiol Rev. (2017) 30:43–113. doi: 10.1128/CMR.00040-16, PMID: 27806994 PMC5217800

[37] Cen M Wang R Kong W Deng H Lei W Chen J. ABO-incompatible living kidney transplantation. Clin Transpl. (2020) 34:e14050. doi: 10.1111/ctr.14050 32713064

[38] Staley EM Carruba SS Manning M Pham HP Williams LA Marques MB. Anti-blood group antibodies in intravenous immunoglobulin may complicate interpretation of antibody titers in ABO-incompatible transplantation. Am J Transplant. (2016) 16:2483–6. doi: 10.1111/ajt.13760, PMID: 26913485

[39] Abbas F El KM Kim JJ Sharma A Halawa A. Thrombotic microangiopathy after renal transplantation: current insights in *de novo* and recurrent disease. World J Transplant. (2018) 8:122–41. doi: 10.5500/wjt.v8.i5.122, PMID: 30211021 PMC6134269

[40] Garg N Rennke HG Pavlakis M Zandi-Nejad K. *De novo* thrombotic microangiopathy after kidney transplantation. Transplant Rev. (2018) 32:58–68. doi: 10.1016/j.trre.2017.10.001 29157988

[41] Bentata Y. Parvovirus B19 in kidney transplantation: key points and essential pitfalls to know. Infect Dis. (2021) 53:404–8. doi: 10.1080/23744235.2021.1893379, PMID: 33641590

[42] Beadle J Papadaki A Toulza F Santos E Willicombe M McLean A. Application of the banff human organ transplant panel to kidney transplant biopsies with features suspicious for antibody-mediated rejection. Kidney Int. (2023) 104:526–41. doi: 10.1016/j.kint.2023.04.015, PMID: 37172690

[43] Roufosse C Simmonds N Clahsen-van GM Haas M Henriksen KJ Horsfield C. A 2018 reference guide to the banff classification of renal allograft pathology. Transplantation. (2018) 102:1795–814. doi: 10.1097/TP.0000000000002366, PMID: 30028786 PMC7597974

[44] Helanterä I Egli A Koskinen P Lautenschlager I Hirsch HH. Viral impact on long-term kidney graft function. Infect Dis Clin N Am. (2010) 24:339–71. doi: 10.1016/j.idc.2010.02.003 20466274

[45] Sharma N Bajwa R. Parvovirus infection-related anemia after kidney transplantation. Case Rep Transplant. (2020) 2020:6437392. doi: 10.1155/2020/6437392, PMID: 32082691 PMC7013336

[46] Thongprayoon C Khoury NJ Bathini T Aeddula NR Boonpheng B Lertjitbanjong P. Epidemiology of parvovirus b19 and anemia among kidney transplant recipients: a meta-analysis. Urol Ann. (2020) 12:241–7. doi: 10.4103/UA.UA_89_19, PMID: 33100749 PMC7546070

[47] Raemer PC Haemmerling S Giese T Canaday DH Katus HA Dengler TJ. Endothelial progenitor cells possess monocyte-like antigen-presenting and t-cell-co-stimulatory capacity. Transplantation. (2009) 87:340–9. doi: 10.1097/TP.0b013e3181957308, PMID: 19202438

[48] Dhanda SK Vir P Raghava GP. Designing of interferon-gamma inducing MHC class-ii binders. Biol Direct. (2013) 8:30. doi: 10.1186/1745-6150-8-30 24304645 PMC4235049

[49] Eid AJ Brown RA Patel R Razonable RR. Parvovirus B19 infection after transplantation: a review of 98 cases. Clin Infect Dis. (2006) 43:40–8. doi: 10.1086/504812, PMID: 16758416

[50] Knysak M Napora M Misiukiewicz-Poć M Pawłowska A Kwella N Zbrzeźniak J. Pure red cell aplasia and antibody-mediated rejection: double trouble in 1 kidney transplant recipient solved by intravenous immunoglobulin infusion: a case report. Transpl Proc. (2020) 52:2530–2. doi: 10.1016/j.transproceed.2020.01.091, PMID: 32276841

[51] Ki CS Kim IS Kim JW Lee NY Kim SH Lee KW. Incidence and clinical significance of human parvovirus b19 infection in kidney transplant recipients. Clin Transpl. (2005) 19:751–5. doi: 10.1111/j.1399-0012.2005.00415.x, PMID: 16313320

[52] Neild G Anderson M Hawes S Colvin BT. Parvovirus infection after renal transplant. Lancet. (1986) 328:1226–7. doi: 10.1016/s0140-6736(86)92245-2 2877370

[53] Bertazza PN Negrisolo S Carraro A Marzenta D Manaresi E Gallinella G. Pre-existing intrarenal parvovirus b19 infection may relate to antibody-mediated rejection in pediatric kidney transplant patients. Int J Mol Sci. (2023) 24:24. doi: 10.3390/ijms24119147, PMID: 37298109 PMC10252308

