# Supplementary material for: ABO-incompatible living donor kidney transplantation failure due to acute blood group antibody-dependent rejection triggered by human parvovirus B19 infection: a case report and literature review
Source: Front Med (Lausanne). 2023 Nov 23;10:1195419. doi: 10.3389/fmed.2023.1195419 (PMC10704095; doi:10.3389/fmed.2023.1195419)
Supplement: Supplementary file 1 [file Presentation_1.PDF]

Supplementary Material

ABO-incompatible Living Donor Kidney Transplantation Failure Due to Acute Blood-group Antibody-Dependent Rejection Triggered by Human Parvovirus B19 Infection: A Case Report and Literature Review

Lin-rui Dai

Correspondence: Sheng Chang: changsheng@hust.edu.cn

Supplementary Figures

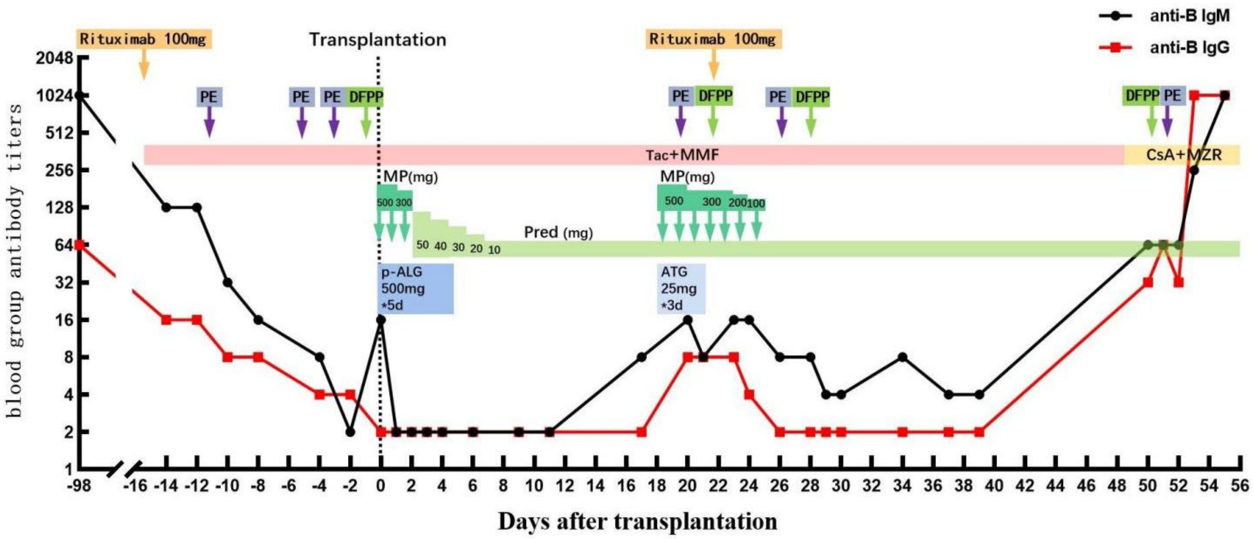

Supplementary Figure 1. Blood group antibody titers and immunosuppressive regimen used before and after transplantation. The recipient's ABO blood group was O and the donor's blood group was B. The recipient's baseline anti-B antibody titers were 1:1024(anti-B IgM) and 1:64(anti-B IgG). recipient completed a pre-transplant desensitization protocol, whereafter, anti-B antibody titers remained at low levels (equal or less than pre-transplant level 1:2) or declined to undetectable level. Until POD 50, the recipient's anti-B IgM and anti-B IgG had increased to 1:64 and 1:32 respectively, and then gradually increased to untreated pre-transplant levels. The pink rectangle indicates tacrolimus (Tac) and mycophenolate mofetil (MMF); The yellow rectangle indicates the change of Cyclosporine (CsA) and mizoribine (MZR) on POD49; The orange arrows indicate days of using rituximab; The purple arrows indicate days of using plasma exchange (PE); The grass green arrows indicate days of using double filtration plasmapheresis(DFPP); The dark green arrows indicate days and dose of using methylprednisolone(MP); The green rectangle indicates days and dose of using prednisone(Pred), staring at 50mg/d and then prednisone tapered by 10 mg every other day to 10 mg/d for maintenance. The dark blue and light blue boxes indicate the dose and days of using Anti-

human T Lymphocyte Porcine Immunoglobulin(p-ALG) and Rabbit Anti-human Thymocyte Immunoglobulin(ATG). The black dashed line indicates the day of transplantation.

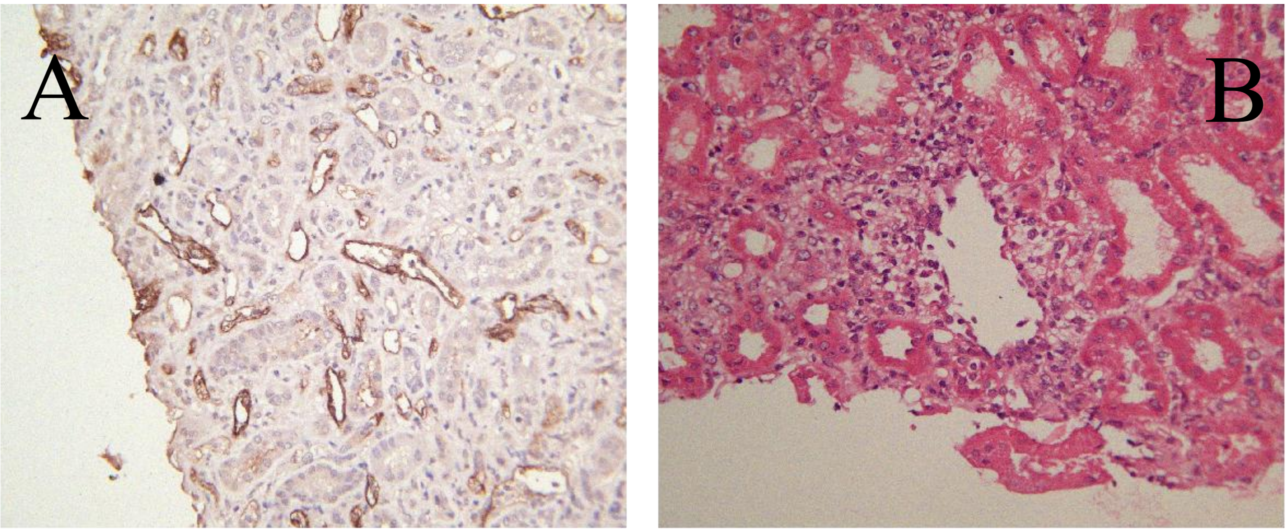

Supplementary Figure 2. Allograft biopsy specimen obtained on POD 18. **(A)** Immunohistochemical staining: C4d was diffuse positive (×200). **(B)** One micro-vein presented with venous endotheliitis; Mild renal interstitial edema, patchy and diffusely infiltration of lymphocytes in the interstitium, patchy and mild renal tubulitis and a few peritubular capillaritis; the interstitial matrix of renal tissue did not show hyperplasia and tubular atrophy; Mild water degeneration of renal tubular epithelial cells, no necrosis of renal tubular epithelial cells. (H.E, ×400)

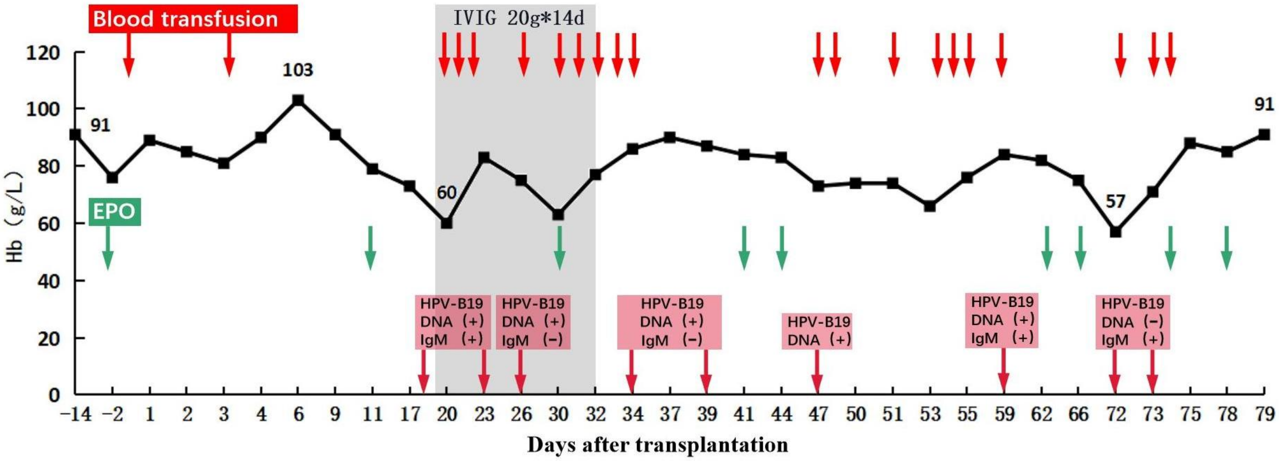

Supplementary Figure 3. Hemoglobin levels before and after transplantation. After transplantation, the recipient's hemoglobin increased to 103g/L at the highest level, and then showed a progressive decrease of hemoglobin without obvious cause, which reached as low as 60g/L on POD 19. After treatment, the recipient hemoglobin (Hb) recovered and remained stable. The red arrows indicate days of using transfusion; the Green arrows indicate days of using recombinant human erythropoietin (EPO); The red squares indicate the results of HPV-B19 DNA and IgM testing and the arrow points to the time of testing.

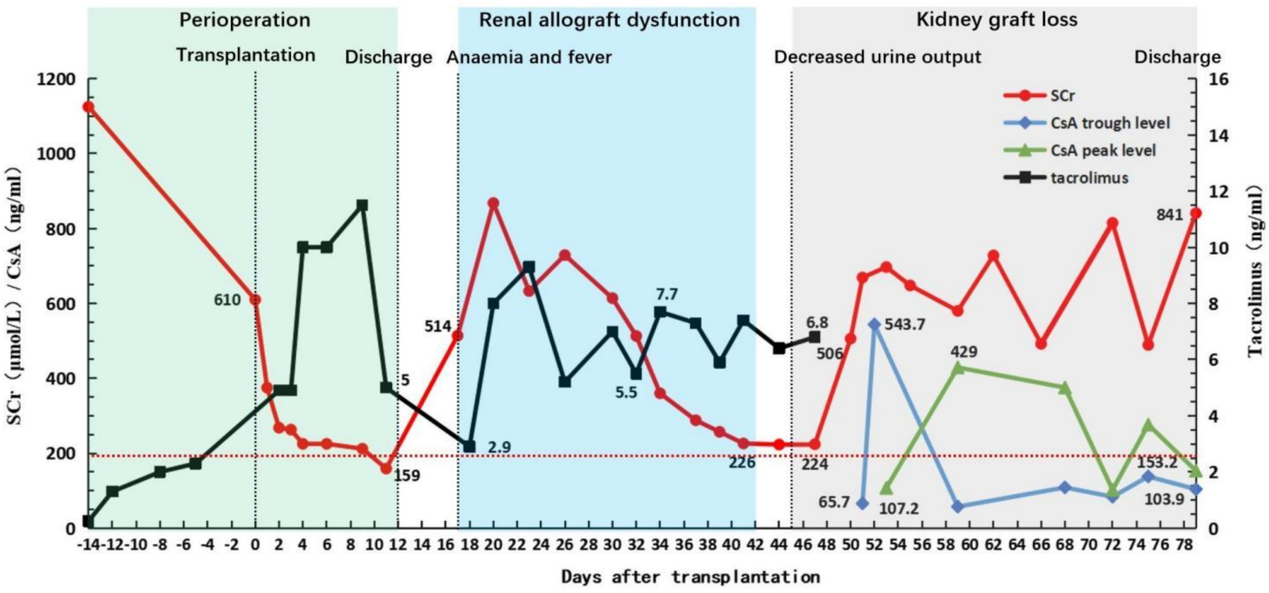

Supplementary Figure 4. Clinical course of the patient. graft function and fluctuation of immunosuppressant concentration before and after transplantation. The green square indicates the patient's first hospitalization and discharges with good recovery of graft function (with serum creatinine (SCr) rapidly declining to 200umol/L within 10 days post-transplantation.); The blue square indicates that the patient was readmitted for treatment due to HPV-B19 infection and aTCMR, after which the patient improved following a series of treatments; The gray square indicates the patient's injury of graft function following a fierce anti-blood group antibody-mediated acute humoral rejection until the graft was lost and the patient was discharged again after resuming regular hemodialysis therapy.

Table 1: Characteristics of the cases of B19V-triggered rejection episodes

Study	Year	Country	Patients (n)	B19V detection	clinical manifestation	renal allograft pathology	The outcome of patients
Barzon et al. (30)	2009	Italy	Kidney transplant patients (7)	PCR	AR (7)<br>fever, rash, and hypo-regenerative anemia (1)	AR (7)<br>High copy numbers of B19V DNA (7)<br>C4d-positive aAMR (1)<br>TMA (1)	Graft survival (6)<br>Acute graft dysfunction (1)
Zolnourian *et al.* (31)	2009	Northern Ireland.	Kidney transplant patients (1)	lgM, lgG and PCR	AR (1)	Acute vascular rejection (1)	Graft failure (1)
MURER *et al.* (35)	2000	Italy	Kidney transplant patients (1)	lgM, lgG and PCR	AR, fever, fatigue and arthralgia, aplastic anemia and thrombocytopenia (1)	TMA (1)<br>Histologic rejection (1)	Graft survival (1)
Eid *et al.* (49)	2006	US	Simultaneous kidney and pancreas transplant patients (1)	lgM, lgG	Chronic rejection, PRCA and leukopenia (1)	Chronic rejection (1)	Graft failure (1)
Knysak *et al.* (50)	2020	Poland	Second kidney transplant patients (1)	lgM, lgG and PCR	AR and PRCA (1)	aAMR (1)<br>acute tubular necrosis (1)<br>BKV infection (1)	Graft survival (1)
Ki *et al.* (51)	2005	Korea	Kidney transplant patients (7)	PCR	AR (7)<br>PRCA (2)	AR (7)	Graft survival (6)<br>Graft failure (1)
Bertazza *et al.* (53)	2023	Italy	Kidney transplant patients (15)	PCR	AR (15)	aAMR (8)<br>aTCMR (7)	Graft survival (15)

NOTE: AR, acute rejection; PRCA, pure red cell aplasia; TMA, thrombotic microangiopathy; aAMR, acute antibody-mediated rejection; aTCMR, acute T cell-mediated rejection.
